# Prevalence and Risk Factors of Self-Medication Among the Pediatric Population in China: A National Survey

**DOI:** 10.3389/fpubh.2021.770709

**Published:** 2022-02-09

**Authors:** Jing Yuan, Wandi Du, Zhiping Li, Qiao Deng, Guo Ma

**Affiliations:** ^1^Department of Clinical Pharmacy and Pharmacy Administration, School of Pharmacy, Fudan University, Shanghai, China; ^2^Department of Clinical Pharmacy, The Children's Hospital of Fudan University, Shanghai, China

**Keywords:** self-medication, self-prescription, survey, pediatric, drug safety

## Abstract

**Background:**

Self-medication of antibiotics is common in China, whereas the self-medication of other medicines is still unknown, especially for the younger children who are vulnerable to adverse drug events. The aim of this study was to estimate the prevalence of self-medication reported by parents among children under age 12 in China.

**Methods:**

A national cross-sectional survey was conducted among parents of children under age 12 in China by using a self-administered online questionnaire. Parents were asked whether they have self-medicated their children in the past 12 months. Logistic regression analysis was performed to access the risk factors of self-medication.

**Results:**

Eligible questionnaires were obtained from 4,608 parents. The majority of respondents were mothers aged between 30 and 39 years old who held a college degree. A total of 1,116 (or 24.21%) respondents reported self-medication in the previous year. In the logistic regression model, parents with graduate degrees were less likely to self-medicate their children [Adjusted OR (AOR) = 0.436; 95% CI = 0.296–0.641]. The odds of self-medication were associated with being a father, living in Northern China, having a child at age 6–11, even though these did not reach statistical significance.

**Conclusions:**

Our findings indicate that self-medication are common in children under age 12, highlighting the drug safety issue in China. It seems that the educational level is the risk factors of self-medication. More targeted intervention and educational program should be implemented to improve drug safety.

## Introduction

Drug safety remains a serious public health issue. The WHO estimates that about 50% of patients fail to take their medicines correctly (1). Every year, nearly nine million children aged under five die worldwide (2), a large part of which is caused by irrational drug use (2). Self-medication or self-prescription by parents is considered as one of the biggest risk factors of drug safety (3). Parental self-medication is common globally, ranging from 7 to 70% (4–8). According to a recent study, one-third of children's population in China had parental self-medication of antibiotics without consultation of physicians or healthcare providers (9). To our best knowledge, however, there is limited data on self-medication of other commonly used medications, such as cold and cough medicines, corticosteroids, and traditional Chinese medicine.

Children are still in the developmental stage, with different pharmacokinetic (PK) and pharmacodynamic (PD) characteristics. Hence, they are more vulnerable to adverse drug events compared to their adult counterparts (10). Due to limited pediatric formula available in the market (11), off-label use of medication is prevalent, ranging from 40 to 90% (12). In China, among 6,020 medicines commonly used in pediatrics, only 238 or 3.95% drugs are approved for pediatric use (13), and most of them are adult formulations with an extended use in children. In addition, the limited availability of pediatric formulations further exaggerates the issue of parental self-medication in China. Considering the adverse outcomes associated with parental self-medication (3, 14), the aim of this study was to estimate the prevalence and the associated risk factors of self-medication among children under age 12 in China.

## Materials and Methods

### Study Design

We employed a cross-sectional design. Parents of children under age 12 were invited to an anonymous online survey, which was available in 34 provinces, autonomous regions, and municipalities which are directly under the central government in China. The study participants were the parents of the children aged under 12 years old. The survey period was from March 2018 to November 2019. This study was approved by the Institutional Review Boards of the Children's Hospital of Fudan University. This study followed the Checklist for Reporting Results of Internet E-Surveys (CHERRIES).

### Survey Questionnaire

The questionnaire, which assessed the prevalence of self-medication use and drug related problems (DRPs), was created based on previous surveys conducted by China Population Communication Center (15). This questionnaire has been developed by an expert panel that consisted of pediatricians, pharmacists, outcomes researchers, and parents. The questionnaire included three sections. The first section asked the demographic and the socioeconomic characteristics of parents, including age, gender, education, and provinces. The second section was to access the prevalence of self-medication for their children. Parents were asked whether their children had been in self-medication in the past 12 months. For those who reported a self-medication for their children, we collected information on the drug classes they commonly used for the self-medication.

### Participant Recruitment

To reach a representative sample of parents, following the previous research (16), we first selected 34 research coordinators to be the original deliverers who invited the parents in their communities to participate. Parents with children aged under 12 were invited to participate. Then, we sent out requests *via* WeChat private messages, including a link to the web-based questionnaire through an internet survey portal (https://www.wjx.cn/). As one of the largest social media platforms in China, WeChat has been used previously to distribute online surveys (17–19). To avoid multiple responses from the same individuals, each WeChat account was only allowed to answer the questionnaire once. A total of 5,189 parents were invited and 4,608 of them completed the survey. The response rate was 88.80%.

The accomplished questionnaire was considered as eligible if (1) all the questions were answered, (2) self-reported age of children was <12, and (3) they were parents who take care of their children. We also excluded questionnaire with the same answers to different questions to ensure the quality of survey.

### Data Analysis

For descriptive analysis, frequency distributions (e.g., percentage) were estimated for categorical variables. Fisher's exact test was used to compare the difference between categorical variables. We also constructed a logistic regression model to examine the potential predictors of self-medication. Both crude and adjusted OR were estimated. Statistical significance was determined at a-level of 0.05. All statistical analyses were performed using SAS 9.4.

## Results

### Characteristics of Respondents

A total of 4,608 (or 88.80%) of 5,189 questionnaires were included in the analysis, after discarding 581 questionnaires that were considered ineligible based on the predefined selection criteria. As shown in Table 1, the majority (*n* = 2,982; 64.71%) of respondents were aged between 30 and 39 years old, 3,563 (77.32%) were mothers, and 2,437 (52.89%) held a college degree. The majority of parents had a child aged between 6 and 11 (*n* = 2,799; 60.74%).

**Table 1:** Characteristics of respondents.

**Characteristics**	**Overall**		**Self-medication**		**No self-medication**		***P*-value**
	**(*****n*** **=** **4,608)**		**(*****n*** **=** **1,116)**		**(*****n*** **=** **3,492)**		
	***n***	**%**	***n***	**%**	***n***	**%**	
**Parent's age**							0.292
LT30	921	19.99	211	18.91	710	20.33	
30–39	2,982	64.71	744	66.67	2,238	64.09	
GT40	705	15.30	161	14.43	544	15.58	
**Relationship**							0.005
Father	1,045	22.68	219	19.62	826	23.65	
Mother	3,563	77.32	897	80.38	2,666	76.35	
**Parent's education**							<0.0001
Middle school	847	18.38	233	20.88	614	17.58	
High school	990	21.48	270	24.19	720	20.62	
College	2,437	52.89	575	51.52	1,862	53.32	
Graduate	334	7.25	38	3.41	296	8.48	
**Regions**							<0.0001
Northeast China	33	0.72	6	0.54	27	0.77	
Northern China	1,794	38.93	603	54.03	1,191	34.11	
Eastern China	1,472	31.94	243	21.77	1,229	35.19	
Southern China	87	1.89	11	0.99	76	2.18	
Central China	302	6.55	66	5.91	236	6.76	
Northwest China	477	10.35	118	10.57	359	10.28	
Southwest China	443	9.61	69	6.18	374	10.71	
**Children's age**							<0.0001
LT1	150	3.26	21	1.88	129	3.69	
1–3	309	6.71	50	4.48	259	7.42	
3–5	1,350	29.30	309	27.69	1,041	29.81	
6–11	2,799	60.74	736	65.95	2,063	59.08	

### Prevalence of Self-Medication

As shown in Table 1, a total of 1,116 (or 24.21%) respondents reported self-medication in the previous year. Respondents who reported self-medication for their children were more likely to be aged between 30 and 39 (66.67 vs. 64.09%), live in Northern China (54.03 vs. 34.11%), and have a child aged 6–11 (65.95 vs. 59.08%). Respondents with graduate degrees were less likely to self-medicate their children (3.41 vs. 8.48%).

**Figure 1:**
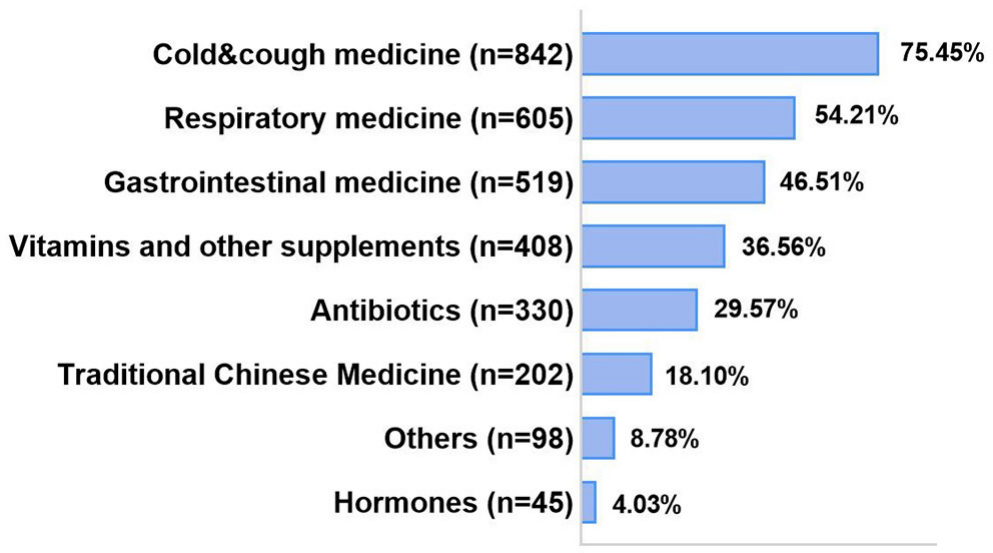
Use of self-medication medicines.

Among those reported parental self-medication, a total of 842 (75.45%) parents reported the use of cold and cough medicine (Figure 1). More than half of respondents reported self-use of respiratory medications (*n* = 605; 54.21%) and gastrointestinal medicine (*n* = 519; 46.51%). A total of 330 (or 29.57%) parents reported self-medication of antibiotics.

### Risk Factors of Self-Medication

In the logistic regression model, the risk factors of self-medication were fathers (Table 2; Crude OR = 1.269; 95% CI = 1.075–1.502), living in Northeastern China (OR = 2.278; 95% CI = 1.001–6.131), and children aged 3–5 (OR = 1.823; 95% CI = 1.155–3.020), and aged 6–11 (OR = 2.192; 95% CI = 1.402–3.599). Parents with college (OR = 0.814; 95% CI = 0.682–0.973) or graduate education (OR = 0.338; 95% CI = 0.231–0.484) were less likely to self-medicate their children.

**Table 2:** Crude and adjusted Odd Ratios (ORs) of having self-medication in the previous year.

**Variables**	**Crude**				**Adjusted**			
	**Estimate**	**OR**	**95% CI**	***P*-value**	**Estimates**	**OR**	**95% CI**	***P*-value**
**Parent's age**
LT30	Ref	Ref	—	—	Ref	Ref	—	—
30–39	0.112	1.119	(0.941–1.334)	0.208	−0.059	0.943	(0.777–1.145)	0.554
GT40	−0.004	0.996	(0.788–1.257)	0.972	−0.225	0.798	(0.618–1.032)	0.085
**Relationship**
Father	0.238	1.269	(1.075–1.502)	**0.005**	0.118	1.125	(0.946–1.339)	0.183
Mother	Ref	Ref	—	—	Ref	Ref	—	—
**Parent's education**
Middle school	Ref	Ref	—	—	Ref	Ref	—	—
High school	−0.012	0.988	(0.805–1.214)	0.910	0.102	1.108	(0.896–1.369)	0.346
College	−0.206	0.814	(0.682–0.973)	**0.023**	−0.086	0.917	(0.756–1.113)	0.382
Graduate	−1.084	0.338	(0.231–0.484)	**<0.001**	−0.831	0.436	(0.296–0.641)	**<0.001**
**Regions**
Northeast China	Ref	Ref	—	—	Ref	Ref	—	—
Northern China	0.823	2.278	(1.001–6.131)	**0.040**	0.583	1.792	(0.726–4.426)	0.206
Eastern China	−0.117	0.890	(0.388–2.404)	0.798	−0.324	0.723	(0.292–1.790)	0.484
Southern China	−0.429	0.651	(0.224–2.046)	0.440	−0.594	0.552	(0.185–1.651)	0.288
Central China	0.230	1.258	(0.530–3.485)	0.626	−0.003	0.997	(0.391–2.543)	0.995
Northwest China	0.391	1.479	(0.636–4.04)	0.399	0.158	1.172	(0.466–2.947)	0.737
Southwest China	−0.186	0.830	(0.352–2.29)	0.692	−0.401	0.670	(0.264–1.701)	0.399
**Children's age**
<1 year	Ref	Ref	—	—	Ref	Ref	—	—
1–3 years	0.171	1.186	(0.692–2.095)	0.545	0.256	1.292	(0.737–2.263)	0.371
3–5 years	0.601	1.823	(1.155–3.020)	**0.014**	0.428	1.534	(0.933–2.520)	0.092
6–11 years	0.785	2.192	(1.402–3.599)	**0.001**	0.398	1.489	(0.903–2.455)	0.119

*The bold font indicates these variables reached statistical significance*.

After adjusting for other risking factors, parents with graduate degrees were less likely to self-medicate their children [Table 2; Adjusted OR (AOR) = 0.436; 95% CI = 0.296–0.641]. The odds of performing self-medication were higher among fathers (OR = 1.125; 95% CI = 0.946–1.339), living in Northern China (OR = 1.792; 95% CI = 0.726–4.426), and having a child at age 6–11 (OR = 1.489; 95% CI = 0.903–2.455), even these did not score statistical significance.

Nearly half of respondents reported misuse of antibiotics (*n* = 1,976; 42.88%), a total of 1,654 (or 35.89%) respondents reported abuse or misuse of cold and cough medicines. The abuse or misuse of traditional Chinese medicine (TCM) was also reported among 24.48% of respondents (*n* = 1,128).

## Discussion

In this large national survey, nearly one-fourth of parents reported self-medication in the previous year, indicating that self-medication is common in children under age 12. Our findings highlight the drug safety issue in China. The risk of self-medication reported by our study is similar to those observed in other countries. The prevalence of parental self-medication was 16.1% in Brazil (5) and 32.8% in France (6), suggesting that parental self-medication is a global issue in both developed and developing countries.

Parents are usually the main caregivers for their children, and are mainly responsible for managing the medication therapy of their children. Their knowledge level and attitude toward drug safety greatly affects whether their children use medications rationally. In this analysis, parents with higher education were less likely to perform self-medication for their children, which was different from other countries. In both Germany and Italy, educated parents were more likely to practice self-medication compared to those with lower education level (20, 21). This discrepancy may be explained by how Chinese parents with higher education are more aware of the importance of drug safety. In addition, our findings also indicate that children living in Northern China had a higher risk of self-medication than those living in other regions, which could be potentially explained by the geographic disparities in the economic development level. It seems that the risk of self-medication increases with the age of children. Particularly, our analysis suggested that children aged 6 to 11 were more likely to be self-medicated by their parents, which was consistent with findings from other countries (7, 22).

In China, antibiotics have been pervasively used for children at home or in the clinical settings. In other developing countries, the prevalence of self-medication with antibiotics was as high as 80% (23, 24), leading to the growing concern of the antimicrobial resistance (AMR). Parental self-medication of antibiotics further increases the risk of developing antimicrobial resistance. The self-medication of antibiotics was more prevalent in rural or less economically developed areas. Since parents play a key role in medication management of their children, targeted intervention should be developed for those with lower socioeconomic status to improve their knowledge on the rational drug use. For parents living in the rural or less developed areas, targeted educational campaign should be offered to improve awareness of rational use of medication.

There are several limitations in this study. First, even though this is a national survey, our findings may not reflect the risk of self-medication among the general population because only a small proportion of parents in China participated in the survey. Second, we cannot exclude the possibility of selection bias because parents with higher literacy level were more likely to participate in the survey. As such, it will be difficult to access the knowledge level of those physicians who failed to respond. Lastly, we only included a couple of predictors for self-medication in the survey. Parental self-medication may be influenced by other factors, such as level of income, non-availability of health care centers, and the number of children.

In conclusion, in this large, national survey in China, parental self-medication is prevalent in children under age 12, highlighting the drug safety issue in China. It seems that educational level is the risk factors of self-medication. More targeted intervention and educational program should be implemented to improve drug safety.

## Data Availability Statement

The raw data supporting the conclusions of this article will be made available by the authors, without undue reservation.

## Ethics Statement

The studies involving human participants were reviewed and approved by Children's Hospital of Fudan University. Written informed consent for participation was not required for this study in accordance with the national legislation and the institutional requirements.

## Author Contributions

JY and GM: concept and design. GM: acquisition, analysis, or interpretation of data. JY: drafting of the manuscript and statistical analysis. JY, WD, and GM: critical revision of the manuscript for important intellectual content. GM, WD, and QD: administrative, technical, or material support. GM and ZL: supervision. All authors approved the final manuscript as submitted and agree to be accountable for all aspects of the work.

## Funding

This research was supported by grants from the National Natural Science Funds of China (82074109, 81873078, 81374051), the Science and Research Program of Shanghai Municipal Health Commission (201740094, 2018YP001), Key Undergraduate Education Reform Projects in Shanghai in 2020 (No. 12), Shanghai Education Science and Research Project in 2019 (C19077) from Shanghai Municipal Education Commission, and Key Innovative Team of Shanghai Top-Level University Capacity Building in Clinical Pharmacy and Regulatory Science at Shanghai Medical College, Fudan University (HJW-R-2019-66-19).

## Conflict of Interest

The authors declare that the research was conducted in the absence of any commercial or financial relationships that could be construed as a potential conflict of interest.

## Publisher's Note

All claims expressed in this article are solely those of the authors and do not necessarily represent those of their affiliated organizations, or those of the publisher, the editors and the reviewers. Any product that may be evaluated in this article, or claim that may be made by its manufacturer, is not guaranteed or endorsed by the publisher.
